# New ultrasound-guided L5 selective nerve root block puncture approach for the treatment of pain due to high-iliac-crest lumbar disc herniation: A case report

**DOI:** 10.1097/MD.0000000000040423

**Published:** 2024-11-15

**Authors:** Weiwei Gao, Jie Zeng, Min Wang, Li Tang, Hao Yang, Yanan Wang, Wei Li

**Affiliations:** a Department of Pain Treatment, Chongqing Hospital of Traditional Chinese Medicine, Chongqing, China.

**Keywords:** case report, in-plane approach technique, L5 nerve root block, pain management, ultrasound guidance

## Abstract

**Rationale:**

Lumbar 5 (L5) selective nerve root block is a common treatment for lumbar 4/5 disc herniation. It is difficult to perform real-time ultrasound-guided targeted L5 nerve root block because of the deep structure of the L5/S1 intervertebral foramen and the occlusion of the sacrum and ilium. Therefore, the safe and efficient implementation of L5 nerve root block is very important for improving the clinical promotion and use of this procedure.

**Patient concerns:**

A 43-year-old male, who presented with a 1-month history of lumbosacral and left lower limb pain.

**Diagnoses:**

The characteristic manifestation of pain symptoms was continuous and distending-like pain, accompanied by numbness on the posterior side of the left lower limb. The pain could be exacerbated by prolonged standing, sedentary behavior, and turning over while being relieved by lying down to rest. The visual analog score was 7 triggered by innocuous stimuli, configuring a clinical picture of typical protrusion of the lumbar intervertebral disc. Physical examination: muscle tenderness, straight leg-raising test of the left lower limb was 60° (+), test of supinating and throwing out one’s belly (+), and Achilles tendon reflex (‐).

**Interventions:**

The patient underwent an ultrasound-guided L5 nerve root block with a modified puncture approach technique called the “transverse process-zygapophysis separation method.”

**Outcomes:**

The patient had a successful nerve blockade characterized by significant reduction in pain after the operation.

**Lesson:**

The innovative puncture approach method may be considered a therapeutic option in patients with chronic pain.

## 1. Introduction

Ultrasound technology has been widely used in spinal-related anesthesia and analgesia techniques, such as prespinal puncture evaluation, local anatomy, and guided puncture in recent years.^[1]^ Ultrasound technology can provide a visual and radiation-free operating platform for real-time, dynamic, and continuous viewing of local anatomical images and improve the accuracy and safety of spinal puncture. Target puncture of the L5 nerve root is a key link in the treatment of lumbar 4/5 disc herniation and herpes zoster neuralgia in the area innervated by the L5 nerve, which can ensure the success of such surgery and treatment. The traditional puncture methods of L5 nerve root block under real-time ultrasound guidance include long-axis out-of-plane puncture and short-axis in-plane puncture. The former is too tough for beginners to master, and the latter is difficult to control the puncture angle due to the existence of facet joint hyperplasia and obstructive ilium. Therefore, there is an urgent need for a puncture technique that can reduce operative difficulty and minimize the damage to nerves and blood vessels. In view of this, we presented a new L5 nerve root puncture approach technique called the “transverse process-zygapophysis separation method” for the treatment of a patient with pain caused by high-iliac-crest lumbar disc herniation compressing the L5 nerve root.

## 2. Case description

A 43-year-old male patient presented with a 1-month history of lumbosacral and left lower limb pain. The patient weighed 57 kg and was 172 cm tall (BMI, 19.3 kg/m^2^). There was no previous medical history. The characteristic manifestation of pain symptoms was continuous and distending-like pain, accompanied by numbness on the posterior side of the left lower limb. The pain could be exacerbated by prolonged standing, sedentary behavior, and turning over while being relieved by lying down to rest. The visual analog score was 7 triggered by innocuous stimuli, configuring a clinical picture of typical protrusion of the lumbar intervertebral disc. Physical examination: muscle tenderness, straight leg-raising test of the left lower limb was 60° (+), test of supinating and throwing out one’s belly (+), and Achilles tendon reflex (‐). Based on the above symptoms and signs, a diagnosis of left-sided L4/5 lumbar disc herniation was made, and an ultrasound-guided L5 nerve root block was planned.

**Figure 1.**
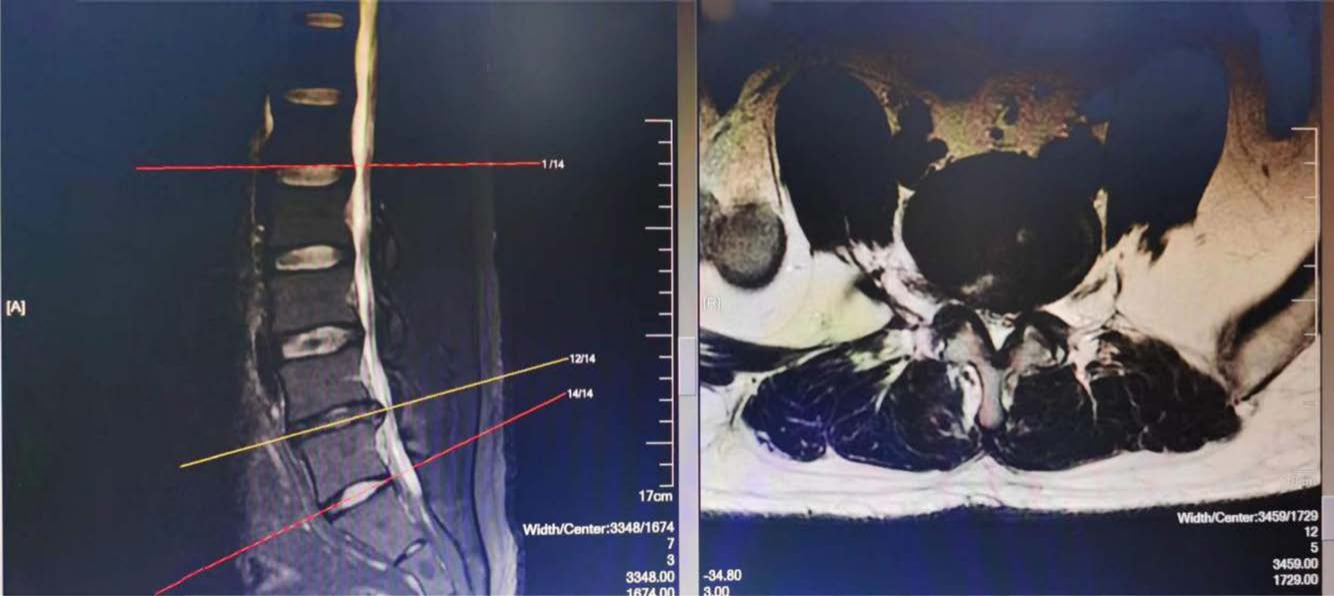
Magnetic resonance imaging of the patient.

Reading photography before the procedure showed that the patient’s iliac crest was high, and the gap between the transverse process of the fifth lumbar and sacrum was significantly narrowed (Fig. 1), which resulted in the inability to achieve conventional in-plane needle insertion. However, out-of-plane needle insertion has difficulty reaching the target, ultimately leading to unsatisfactory therapeutic effects. After consultation among the doctors, oblique section scanning at an oblique angle to the spine was determined.

**Figure 2.**
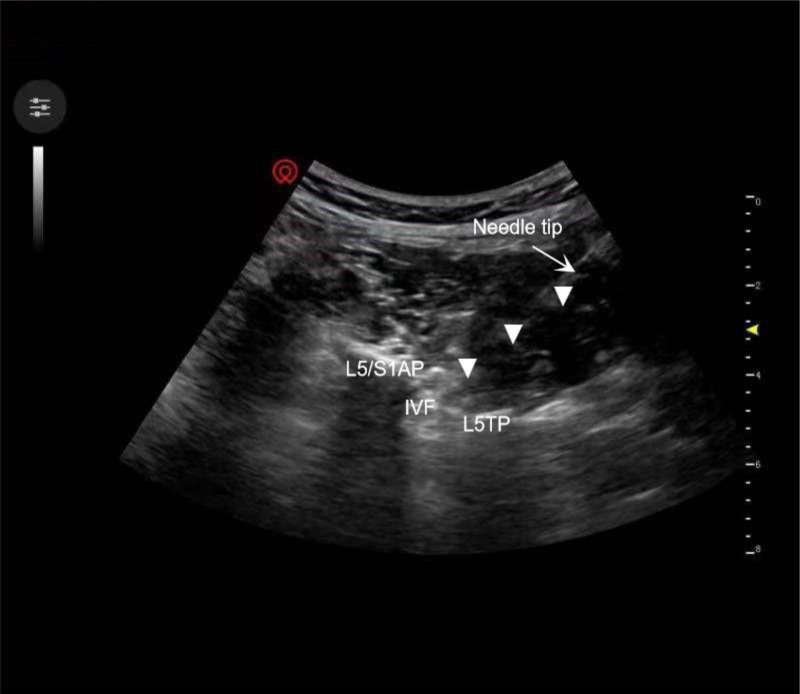
Ultrasound imaging showing transverse process-articular process separation. IVF = Intervertebral foramen, L5/S1AP = L5/S1 articular process, L5TP = L5 transverse process.

A low-frequency convex array probe (L1–5, navii) was placed transversely between the posterior superior iliac spine and the midline of the spine. The specific method was moving the probe up in parallel. When the ultrasound probe reached the L5 transverse process, the L5/S1 zygapophyseal joint and the root of the L5 transverse process could be observed on the ultrasound image after rotating the probe. At this point, the operator placed one end of the ultrasound probe to the root of the L5 transverse process and the other end to the L5/S1 zygapophyseal joint. Then, the probe was gradually moved from the inside to the outside. The probe was then moved laterally, and the transverse process and vertebral body could be seen gradually separating on ultrasound imaging, which is called the “transverse process-articular process separation sign” (Fig. 2). After the position was determined, real-time guidance was performed using the in-plane technique. When the L5/S1 zygapophyseal joint was reached, the needle was inclined upward and slid along the ventral zygapophyseal joint to the intervertebral foramen.

**Figure 3.**
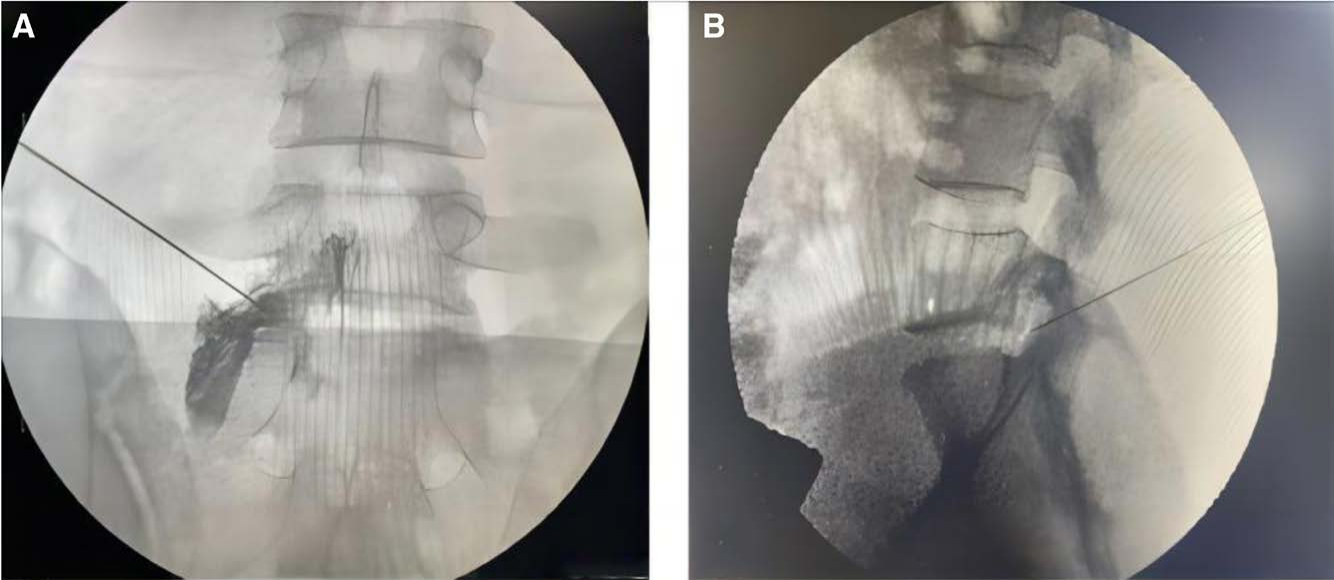
Visualization of the needle reaching the L5 nerve root through the intervertebral foramen under C-arm fluoroscopy.

During the operation, the patient remained in the prone position, with a soft pillow placed below the abdomen to enlarge the lumbar space, and the operator stood on the affected side of the patient. After disinfection and placing a disposable surgical hole towel, the operator held the 21-gauge needle in the dominant hand and the probe in the contralateral hand. The in-plane technique was used to perform real-time transforaminal puncture under real-time ultrasound guidance in this section. Puncture the needle from the head to the caudal end, from the dorsal side of the transverse process of L5 to the location of the “separation sign” into the intervertebral foramen. Then, the puncture was in place, and C-arm fluoroscopy was used to show that the needle reached the intervertebral foramen (Fig. 3).

After the operator pulled back to confirm the absence of blood and cerebrospinal fluid, iohexol injection (*SFDA approval number: H19980218*, *Beijing Beilu Pharmaceutical Co*., *Ltd*., Beijing) 2 mL was injected. When the L5 nerve root was developed by C-arm fluoroscopy, a mixture of 2% lidocaine (*SFDA approval number*: *H13022313*, *Hebei Tiancheng Pharmaceutical Co*., *Ltd*., Cangzhou) and dexamethasone palmitate injection (*SFDA approval number: HJ20160168*, *Mitsubishi Tanabe Pharma Co*., *Ltd*., Chuo-ku) was injected into the foramen. The patient’s vital signs were stable during the operation, and he had a successful nerve blockade characterized by obvious reduction in pain after the operation (The visual analog score was 2 scores on the second day after surgery and 2 scores after 1 week), and the pain was relieved completely at the follow-up 1 month later.

**Figure 4.**
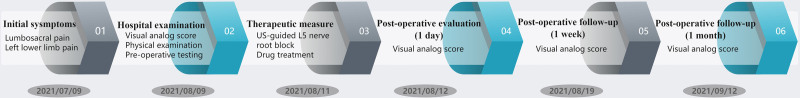
The timeline of the clinical course.

Moreover, the patient was treated with 0.4 mg of Bulleyaconitine A tablets (*SFDA approval number*: *H20057421*, *Yunnan HAOPY Pharmaceutical Co*., *Ltd*., Kunming) 3 times per day and 1 patch of Flubiprofen cataplasms (*SFDA approval number: H20103549*, *Tide Pharma Co*., *Ltd*., Beijing) per day for 7 consecutive days. And at the last follow-up, the patient indicated that he did not need to undergo any further checking and did not feel it was necessary to pursue other rehabilitation due to a sufficient pain relieve. The patient showed good tolerance and compliance with the interventions, and no adverse events occurred throughout the course of treatment or during follow-up period. Figure 4 illustrates the timeline of the clinical course.

## 3. Discussion

Lumbar disc herniation is a common clinical disease that is mainly characterized by lumbosacral pain and often accompanied by lower limb numbness.^[2]^ The lower lumbar spines are the most predilection segments of lumbar disc degeneration, and approximately 95% of lumbar disc herniation occurs in the intervertebral discs of L4/5 and L5/S1.^[3]^ L4/5 disc herniation is often associated with pain, which is caused by compression of the L5 nerve. Selective L5 nerve root block is an important treatment for pain caused by L5 nerve root entrapment.^[4]^ However, the most difficult task in L5 nerve root puncture lies in the particularity of the anatomical structure. The L5 nerve root travels in the lumbosacral tunnel, exits the intervertebral foramen, and moves across the sacral wing into the pelvis outward and downward. The lumbosacral tunnel is composed of the L5 transverse process, sacrum, lumbosacral ligament, and intertransverse ligament.^[5]^ Due to the narrow space of the lumbosacral tunnel, there may be some difficulty in performing the procedure at this level. In this case, if there are anatomical variations and other abnormal conditions at the same time, such as sacralization of the lumbar vertebra, lumbarization of the sacral vertebra, and obvious narrowing of the gap between the L5 transverse process and the sacrum,^[6]^ it is difficult to insert the puncture needle, ultimately leading to a decrease in the accuracy of the puncture approach.

At present, 3 approaches have been proposed for selective L5 nerve root block under US guidance. Sato et al^[6]^ used a long-axis view to scan the base of the transverse process 3 cm from the midline and then performed in-plane puncture to the L5 nerve root, which presented a hyperechoic linear structure below the transverse process. However, this approach may affect the success rate of puncture due to the wide transverse process and the narrow space surrounding the sacrum. Kim et al^[7]^ proposed using the transverse processes under the longitudinal view as an ultrasound landmark, and the cephalad and caudal medial branch block to the nerve root was undertaken in the transverse view of the sonogram as a short axis in the plane approach. Then, another needle of length equal to that for the medial branch block was inserted for nerve root block between the 2 transverse processes under the longitudinal view. The downside of this approach is that the clarity of the spine structure on US images can be compromised by the high acoustic impedance of bone, and it is difficult to perform and reproduce. Nathan et al^[5]^ valued the relationship between the “lumbosacral tunnel” and the patient’s L5 nerve root compression and advocated the use of the “lumbosacral tunnel” method for L5 nerve root block under ultrasound guidance. After the probe was placed perpendicular to the iliac crest and gradually translated to the posterior superior iliac spine, the acoustic shadow of the L5 transverse process and iliac crest and the lumbosacral tunnel between the 2 hyperechoic lines were observed. An out-of-plane puncture into the lumbosacral tunnel was then performed for the L5 nerve root block under ultrasound guidance. However, this method may lead to a poor puncture effect due to the variation in local anatomical structure.

**Figure 5.**
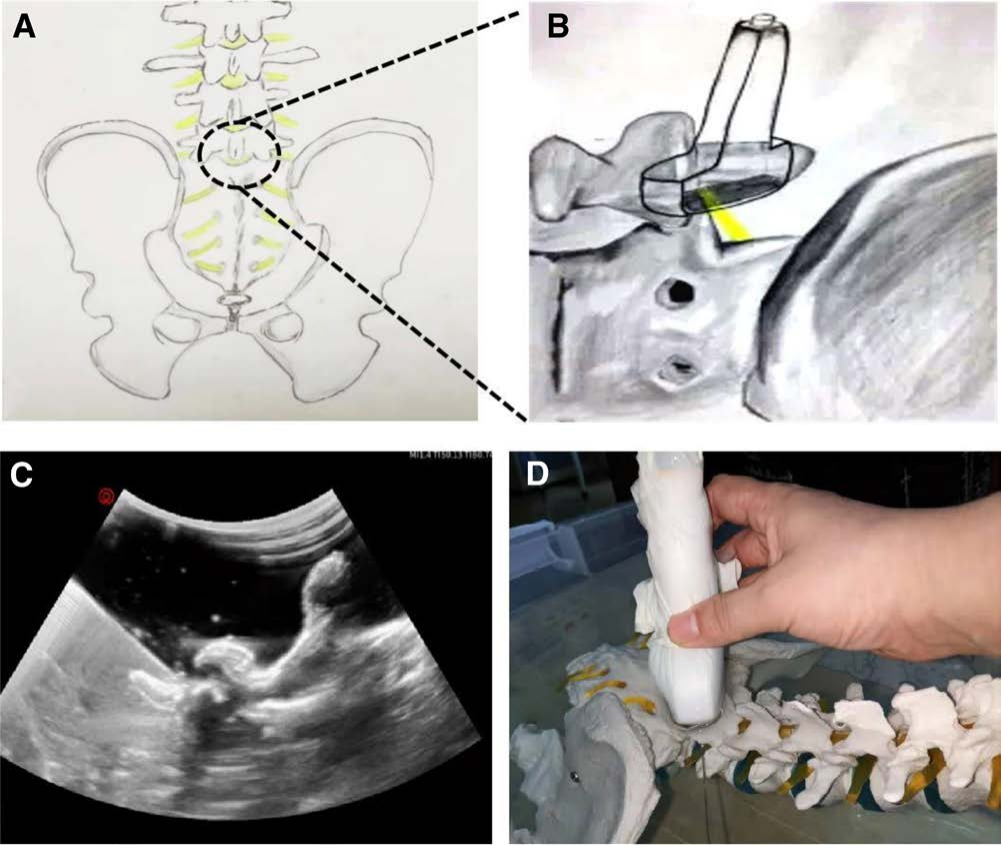
Water box experiment. (A) Schematic showing the site of the “transverse process-vertebral separation sign.” (B) Schematic shows the direction in which the probe moves (one end of the ultrasound probe is placed at the root of the L5 transverse process and the other end to the L5/S1 zygapophyseal joint). The probe was gradually moved from the inside to the outside. (C) Under ultrasound guidance, the needle tip was inserted between the “transverse process-vertebral separation sign.” (D) Puncture direction on the model.

Therefore, we tried to improve the technique of L5 nerve root puncture under US guidance, that is, using the “transverse process-zygapophysis separation method” to perform in-plane puncture. One end of the ultrasound probe was placed at the root of the L5 transverse process and the other end to the L5/S1 zygapophyseal joint. The probe was gradually moved from the inside to the outside, and the transverse process and the vertebral body were gradually separated so that they could be observed on ultrasound imaging. We explained this phenomenon as the “transverse process-vertebral separation sign” through a water box experiment simulated by plastic models (Fig. 5). An in-plane puncture was performed when the “transverse process-vertebral separation sign” appeared, and the needle was punctured from the head end to the caudal end and from the dorsal side of the L5 transverse process. We found that the L5 transverse process can shield-like cover the L5 nerve root, which is located at the ventral side of the transverse process, thus successfully and effectively avoid nerve injury and aid injection into the vein. Moreover, the lumbosacral tunnel could be displayed as much as possible on the oblique plane, which successfully avoided the obstruction of the high iliac crest and increased the operating space. During the in-plane puncture, the needle could be displayed throughout the lumbosacral tunnel and reach the ventral surface of the zygapophysial joint. Then, the beveled side of the needle was slowly slid into the foramina to avoid injury to the L5 nerve root to achieve the treatment of L5 intervertebral foramen injection.

## 4. Conclusion

We believe that the new technique described by us provides advantages over the method described by the studies listed above. Our technique not only improves the efficiency and safety of the lumbar transforaminal puncture technique but also shortens the learning curve for beginners to understand and master this technique. This innovative technique will become a beneficial complement for L5 nerve root puncture and is suitable for clinical popularization. Of course, randomized controlled trials need to be designed and conducted in the future to further verify the safety and effectiveness of this modified approach technique.

## Acknowledgments

We thank all involved in this report.

## Author contributions

**Data curation:** Min Wang, Li Tang, Hao Yang.

**Writing – original draft:** Weiwei Gao, Jie Zeng.

**Writing – review & editing:** Yanan Wang, Wei Li.
